# Comparison study of EMG signals compression by methods transform using vector quantization, SPIHT and arithmetic coding

**DOI:** 10.1186/s40064-016-2095-7

**Published:** 2016-04-12

**Authors:** Eloundou Pascal Ntsama, Welba Colince, Pierre Ele

**Affiliations:** Physics Department, Faculty of Sciences, University of Ngaoundere, PO Box 454, Ngaoundere, Cameroon; Department of Basic Science, Law and Humanities, Institute of Mines and Petroleum Industries, University of Maroua, PO Box 46, Maroua, Cameroon; Electrical Engineering and Telecommunications Department, National Advanced School of Engineering, University of Yaounde 1, Yaoundé, Cameroon; IUT of the University of Douala, PO Box 8698, Douala, Cameroon

**Keywords:** Compression, EMG, DWT, DCT, Vector quantization, SPIHT, Arithmetic coding

## Abstract

In this article, we make a comparative study for a new approach compression between discrete cosine transform (DCT) and discrete wavelet transform (DWT). We seek the transform proper to vector quantization to compress the EMG signals. To do this, we initially associated vector quantization and DCT, then vector quantization and DWT. The coding phase is made by the SPIHT coding (set partitioning in hierarchical trees coding) associated with the arithmetic coding. The method is demonstrated and evaluated on actual EMG data. Objective performance evaluations metrics are presented: compression factor, percentage root mean square difference and signal to noise ratio. The results show that method based on the DWT is more efficient than the method based on the DCT.

## Introduction

Electromyography has a great important in pathological diagnostic, of patients suffering of neuromuscular disorders and for the prevention of premature births; well many data are recorded and stored in the hospitals. These data can be sent to another health center for diagnosis by a specialist; and thus arises the problem of storage and transmission. With the development of telemedicine, the storage problem and transmission of biomedical signals has become a top priority. Compression is an alternative to solve this problem. In the literature, we have two types of compression: lossless compression that gives a good signal reconstruction but which hardly yields high compression ratio and lossy compression which often includes quantification stage to improve the compression ratio. Compression of EMG signals already been the subject of some work and we have the development of new techniques and compression formats. According to the works of Sana and Kaïs (2009) a recording of an electrocardiogram (ECG) per day at a resolution of 12 bits/sample requires to average of over 100 megabytes of memory. These numbers far exceed the capabilities of traditional systems of storage and transmission. The literature of EMG signals compression (especially surface EMG) echoes several techniques and methods. The compression of EMG signals using the Embedded Zero Tree Wavelet has been studied with compression Factor in the range 60–95 % by Norris et al. (2001). The algorithms for EMG signal compression using wavelet transform, and a scheme for the dynamic allocation of the bits that represent wavelet coefficients have been proposed by Berger et al. (2006, 2007). In the works of Carotti et al. (2005), we have the EMG signal compression technique based on autoregressive (AR) modeling. This technique provides a high compression factor (over 97 %) but it is not applicable if the shape of the signal waveform has to be preserved after compression. Discrete wavelet packet transform with optimization of the mother wavelet and wavelet packet basis were used for compression of biomedical signals (Brechet et al. 2007). The same year, Jain and Vig (2007) proposed EMG compression method based on vector quantization combined with wavelets. The year 2008 was marked by the work of Paiva et al. (2008) who proposed adaptive EMG compression using optimization wavelet filters. The work of Filho et al. (2008) adopted the multiscale multidimensional parser algorithm. The works of Carotti et al. (2008) and Marcus et al. (2009) applied to the EMG signals, techniques for image compression and gets a compression factor of the order of 80 % with a PRD from 3.82 to 4.43 %. In the more recent work of literature, we can found the works of Trabuco et al. (2013), such as “Compression of EMG signals by Transforms and Spectral Profile for Bit-Allocation” and “S-EMG signal compression based on domain transformation and spectral shape dynamic bit allocation” (Trabuco et al. 2014). In this paper, we make a comparative study between DCT and DWT for compression of EMG signals, using vector quantization which are associated SPIHT coding and arithmetic coding. In this work, lossy compression is exploited. We propose a new algorithm for the EMG signal compression. The performances of this method under study are determined by the PRD, signal to noise ratio, the compression factor and the subjective criteria.

## Background

**Fig. 1:**
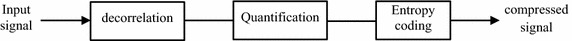
Lossy compression scheme

Compression systems which can guarantee high compression ratios operate according to Fig. 1.

These compression systems concern lossy compression methods; that exploit at best the redundancy in the signal. Most of these compression systems are using transformed methods, which allow switching from spatial domain to a transform domain where the coefficients are low correlation. This step is carried out by a mathematical transformation followed by quantization step. The final step of these systems is entropy coding which produces the bit stream representing the compressed data.

Two transforms were used by the new compression approach proposed: the discrete cosine transform and the discrete wavelet transform. These transforms are used at the decorrelation. The decorrelation extracts the relevant signal information and reduces redundancy in the signal. Most decorrelators are based on reversible transformation. The principle of these decorrelators, consist to focus the information on a small number of values, the other being near zero.

The purpose of processing is to project the signal on a basis function whose properties are adapted to the nature and characteristics of signal to be analyzed. The projection is orthogonal in order to guarantee a decorrelation of obtained coefficients (Gaudeau 2006).

### Theory of wavelet transform and discrete cosine transform

The wavelet transform of a signal x(t) can be defined as the projection on the basis of wavelet functions:

1 $$TOD\left( {a,b} \right) = \frac{1}{\sqrt a }\int_{ - \infty }^{ + \infty } {x\left( t \right)} \varPsi \left( {\frac{t - b}{a}} \right)dt,\quad {\text{avec}} \in {\text{R}},\quad {\text{a}} \ne 0$$

2 $$TOD\left( {a,b} \right) = \int_{ - \infty }^{ + \infty } {x\left( t \right)} \varPsi_{a,b} \left( t \right)dt,\quad {\text{avec}}\;\varPsi_{a,b} \left( t \right) = \frac{1}{\sqrt a }\varPsi \left( {\frac{t - b}{a}} \right)$$

The functions $\varPsi_{a,b} \left( t \right)$ are obtained from the dilation and translation of the mother wavelet $\varPsi \left( t \right).$ The functions $\varPsi_{a,b} \left( t \right)$ are sometimes called wavelets girls.

The wavelet transform is reversible.

3 $$x\left( t \right) = \frac{1}{{C_{\varPsi } }}\int_{ - \infty }^{ + \infty } {\int_{ - \infty }^{ + \infty } {\frac{1}{{a^{2} }}\left\langle {\left. x \right|\varPsi_{a,b} } \right\rangle \varPsi_{a,b} \cdot da \cdot db} }$$

4 $${\text{where}}\;C_{\varPsi } = 2\pi \int_{ - \infty }^{ + \infty } {\left| {\tilde{\varPsi }\left( \omega \right)} \right|^{2} } \frac{d\omega }{\omega }$$

$\tilde{\varPsi }\left( \omega \right)$ is the Fourier transform of $\varPsi \left( t \right).$

The wavelet function must check the eligibility requirement:

If $$\varPsi \left( t \right) \in L^{2} ,$$ then:

5 $$\int_{ - \infty }^{ + \infty } {\frac{{\left| {\tilde{\varPsi }\left( \omega \right)} \right|}}{\left| \omega \right|}d\omega < \infty }$$

This condition helps analyze and reconstruct the signal without loss of information. A method for calculating the wavelet transform is to convolve the signal with a pair of quadrature mirror filters selected for a sub-sampling factor of 2 or decimation. These filters that decompose the signal consist of a low-pass filter h and a high pass filter g. They thus divide the bandwidth of the signal exactly in the middle. The coefficients are recombined to synthesize the signal x(t) by the inverse wavelet transform. It is obtained using an over-sampling operation.

Here, the EMG signal is converted into a two-dimensional signal to undergo the image decomposition into sub-bands with different filters (low pass h and high pass g). This requires the use of a separable two-dimensional DWT (lines + columns). The input image is decomposed each time into four sub-images (Image approximated, horizontal detail, vertical detail and diagonal detail) with different low-pass filters and high pass. Reconstruction will be done using quadrature mirror filters, represented by their impulse responses (h and g).

The discrete cosine transform is an orthogonal linear transformation. It is considered a simplified version of the discrete Fourier transform. The transform coefficients are not complex, but real; which is advantageous for the coding and quantization.

The two dimensional discrete cosine transform an image $S_{yx}$ is defined by:

6 $$F_{vu} = \frac{1}{4}C_{v} C_{u} \mathop \sum \limits_{y = 0}^{N - 1} \mathop \sum \limits_{x = 0}^{N - 1} S_{yx} Cos\left( {v\pi \frac{2y + 1}{2N}} \right)Cos\left( {u\pi \frac{2x + 1}{2N}} \right)$$

and the inverse transform is defined by:

7 $$S_{yx} = \frac{1}{4}\mathop \sum \limits_{v = 0}^{N - 1} \mathop \sum \limits_{u = 0}^{N - 1} C_{v} C_{u} F_{vu} Cos\left( {v\pi \frac{2y + 1}{2N}} \right)Cos\left( {u\pi \frac{2x + 1}{2N}} \right)$$

$$\begin{aligned} {\text{with}}\;C_{u} & = \left\{ {\begin{array}{*{20}l} {\frac{1}{\sqrt 2 },} \hfill & \quad {if\, u = 0 } \hfill \\ {1,} \hfill & \quad {else } \hfill \\ \end{array} } \right., \\ C_{v} & = \left( similar\,to\,the\,above \right) \\ \end{aligned}$$

This transform uses a fixed transform matrix whose bases vectors are close to the class of matrices to which belongs the Karhunen–Loeve transform (KLT) (Allen and Bellian 1993).

The compression method of the EMG signal is based on the two-dimensional discrete cosine transform. The 2D DCT is of great interest that has already shown its effectiveness. It is widely used and popular for image coding, as shown its adoption by the JPEG international standard for still image compression.

### Quantization and coding

To quantify the coefficients from the decorrelation, vector quantization has been exploited.

**Fig. 2:**
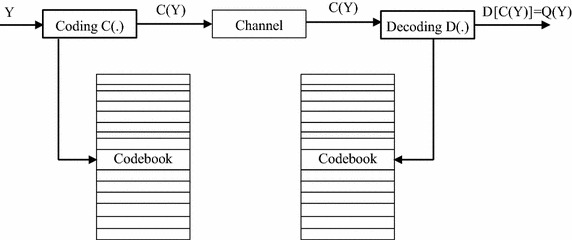
General scheme of a vector quantization

Vector quantization is a generalization of the scalar quantization. It can be seen as a combination of two functions: an encoder and a decoder. The encoder is for any vector Y of the input signal, to look in the codebook vector Y to the nearest code. It is only the address of the vector Y and the selected code which will be transmitted. The decoder has a replica of the codebook and consults it to provide the code vector index corresponding to the received address. Vector quantization is represented by Fig. 2.

A codebook plays an important role in Vector Quantization, which consists of collection of code vectors. The literature presents many algorithms for generating the codebook: Linde, Buzo and Gray (LBG) (Jain and Vig 2007; Shaou et al. 2002; Gronfors and Paivinen 2005; Gronfors et al. 2006), K-Means, Kohonen and the learning algorithm to competition (AC). In this article, we use the algorithm of K-means. The codebook size used is a power of 2. In our work, we tested the size of codebook $2^{5} , 2^{6} , 2^{7} , 2^{8}$ on EMG signals. All these codebooks tested, only the codebook size $2^{5}$ presented a faithful reconstruction of the EMG signals.

The coding has an important place in the compression. The SPIHT coding and the arithmetic coding are operated. The SPIHT coding algorithm is one of the most widely used algorithms in the field of compression. It has been proposed by Said and Pearlman (1996) for encoding the wavelet coefficients; and has been used for the compression of other types of data such as ECG signals (Tai et al. 2005; Lu et al. 2000) and video signals (Pearlman et al. 1998). The SPIHT algorithm (Said and Pearlman 1996) instructs partially information while adding some extra information. This algorithm provides an improvement of the EZW algorithm (Shapiro 1993) while retaining the properties which are:

- good performance;
- if the product bit stream is interrupted or truncated, the reconstruction of the image is partially possible.

SPIHT is based on a partial ordering by amplitude via a sorting algorithm of partitions, and exploiting similarity present at different levels of the image wavelet transform.

In the SPIHT algorithm, three symbols, namely zerotree (ZT), insignificant pixel (IP) and significant pixel (SP) are used to code the wavelet coefficients of an image, which are stored in the list of insignificant sets (LIS), list of insignificant pixels (LIP) and list of significant pixels (LSP), respectively. The SPIHT coding (Said and Pearlman 1996; Gutzwiller et al. 2009) that we used has been slightly modified on its value $S_{n} \left( {Y_{i} } \right).$

8 $$S_{n} \left( {Y_{i} } \right) = \left\{ {\begin{array}{*{20}l} 1 \hfill & \quad { if\, \left| {Y_{i} } \right| \le \frac{{2^{n} }}{n}} \hfill \\ 0 \hfill & \quad {if\, \left| {Y_{i} } \right| > \frac{{2^{n} }}{n}} \hfill \\ \end{array} } \right.$$

With *n* = *|log*_2_(*max*_*i*_*|Y*_*i*_|)| where $0 \le i \le \;{\text{n}},$, the number of coefficients to encode and $S_{n}$ the importance of pixel $Y_{i}$ as approximation or detail and the profit is that each part of our image can be considered as a detail or not according to threshold value.

The Arithmetic coding allows, from the probability of occurrence of the symbols of a source to create a single code word that is associated with a sequence of arbitrary length symbols. This differs from the Huffman encoding that assigns code words to variable lengths to each source symbol. The associated code with a sequence is a real number in the interval [0, 1]. This code is built by recursive subdivision of intervals. A range is divided for each new symbol belonging to the sequence. Is obtained, ultimately, a subinterval of the interval [0, 1] such that every real number belonging to this interval represents the sequence to coded. The arithmetic coding principle can be found in Witten et al. (1987).

### Compression approach method

**Fig. 3:**
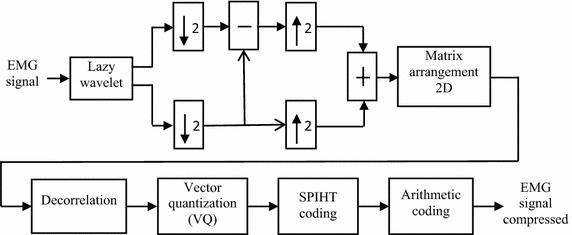
Compression scheme

The new compression approach is proposed through the Fig. 3.

It is composed of a preprocessing block, a decorrelation block, of the vector quantization, a SPIHT coding block and followed by another arithmetic coding block.

The first function used is a separating wavelet whose purpose is to divide the EMG signal into two sub signals. These two sub signals correspond to samples of even indexes and odd indexes. The oversampling of the difference of even and odd index is associated with the even index samples. This step can be regarded as a sub-sampling of input signal and allows us to remove correlation on EMG signal. The operation is done on a part of the input signal. This is to reduce redundancy.

The resulting signal is converted into a 2D signal. For the transformation of EMG signal coefficients in two dimensions, the work of Marcus et al. (2009), Costa et al. (2009), Ntsama et al. (2013) have been exploited, where the coefficients of the EMG signal is divided in *M*_*i*_ sequences multiple of 128, then align each after other and completed with zeros if necessary. The objective is to achieve a 2D matrix. The two-dimensional EMG signal coefficients obtained is divided into 32 × 32 block, in order to reduce noise and errors over a large portion of the signal. The 2D DWT and 2D DCT are used at the decorrelation. We have two compression schemes. Namely: a compression scheme with 2D DWT and another compression scheme with 2D DCT. The different coefficients from the decorrelation are quantified by a vector quantization. The quantized coefficients are encoded doubling by the SPIHT coding and arithmetic coding. This way of proceeding allows increasing the compression ratio.

### Performance parameters used to evaluate compression

The performance of compression algorithms are evaluated from three objective parameters: the compression factor (CF) defined by Eq. (9), the percentage root mean square difference (PRD) given by Eq. (10) and the signal to noise ratio (SNR) given by Eq. (11). These criteria were used in most of the compression articles EMG signals (Norris et al. 2001; Berger et al. 2006, 2007; Paiva et al. 2008; Filho et al. 2008; Marcus et al. 2009; Ntsama et al. 2013; Trabuco et al. 2013, 2014).

9 $$CF = \frac{{EMG_{orig} - EMG_{com} }}{{EMG_{orig} }} \times 100\;\%$$

where $EMG_{orig}$ and $EMG_{com}$ are the original and the compressed file lengths, respectively.

10 $$PRD = \frac{{\sqrt {\mathop \sum \nolimits_{n = 1}^{k} \left( {EMG_{org} \left[ n \right] - EMG_{rec} \left[ n \right]} \right)^{2} } }}{{\sqrt {\mathop \sum \nolimits_{n = 1}^{k} \left( {EMG_{org} \left[ n \right]} \right)^{2} } }}$$

where $EMG_{org} \left[ n \right]$ is the original signal and $EMG_{rec} \left[ n \right]$ is the reconstructed signal and k is the length of the EMG signal.

11 $$SNR = 10log\left( {\frac{{\sigma_{org}^{2} }}{{\sigma_{err}^{2} }}} \right)$$

where $\sigma_{org}^{2}$ is power of original signal and $\sigma_{err}^{2}$ is power of error between the original EMG signal and the reconstructed EMG signal.

EMG signals were collected from the biceps muscle of 4 male subjects (Age: 23–28 years). All subjects were placed in an isometric brace and the forearm was fixed at 90°, maintaining 60 % of their maximum voluntary contraction. All signals were sampled at 2048 Hz, quantized with 12 bits. The EMG signals were amplified (−3 dB, bandwidth: 5–512 H) with a gain of 2000. The duration of the signals varies from 3 to 5 min. Four EMG signals called Kheir1, Kheir2, Jouve3 and EMG_Healthy were used. In the quantization phase, and to find the optimal size of the codebook, we tested each codebooks previously built on the four EMG signals. It emerges from this experiment that the size 25 of the codebook gives a good EMG signal reconstruction. However, the second experiment consisted in looking for a codebook able to encode and decode all four signals effectively. It occurs that the codebook built with the EMG_Healthy signal is able to reconstruct “faithfully” the four EMG signals.

## Results

**Fig. 4:**
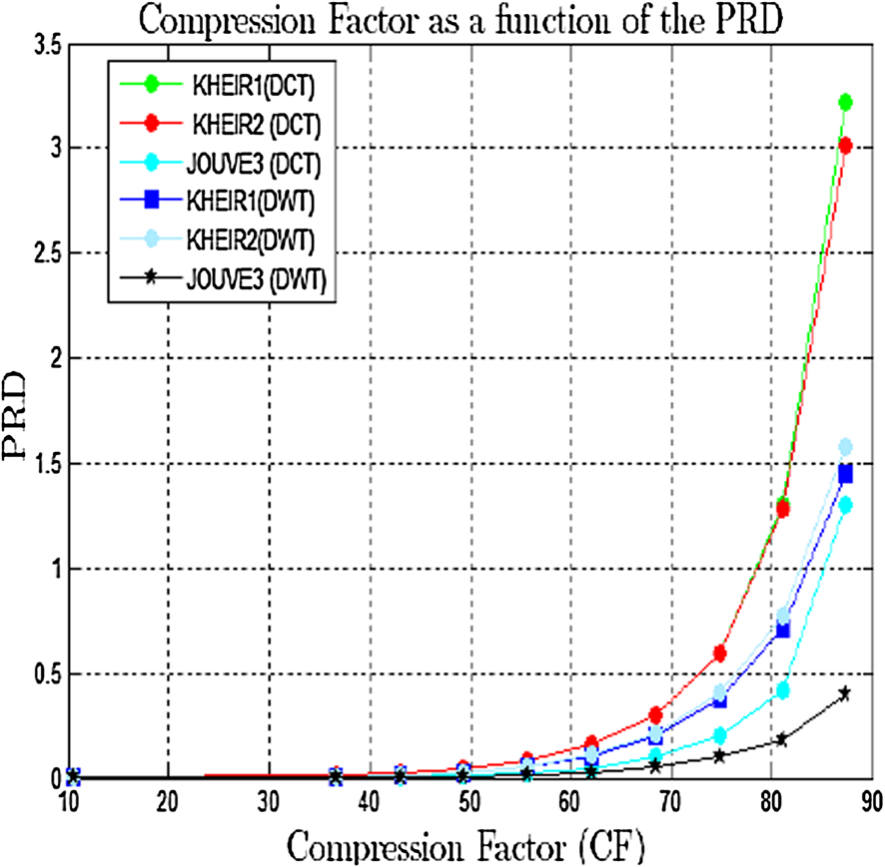
Compression factor as a function of the PRD

Figure 4 shows the variation of the PRD as a function of the compression factor for different methods transform (DCT and DWT) and for different EMG signals.

**Fig. 5:**
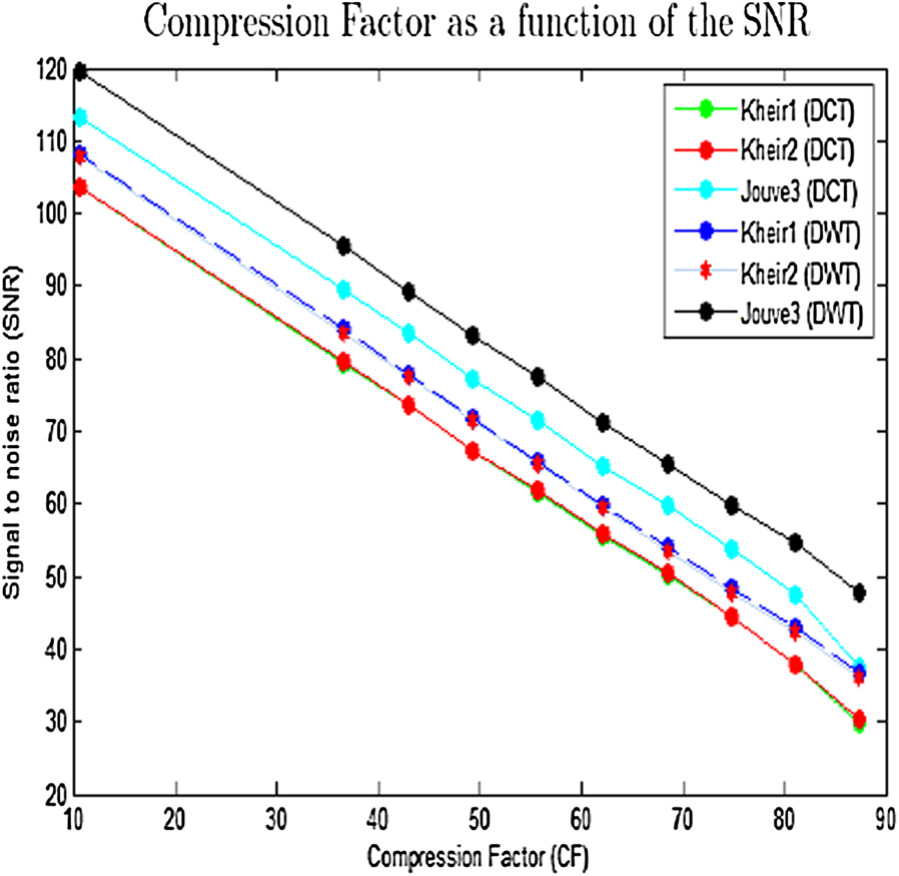
Compression factor as a function of the SNR

Figure 5 give the signal to noise ratio (SNR) as a function of the compression factor for different methods transform (DCT and DWT) and for different EMG signals.

**Fig. 6:**
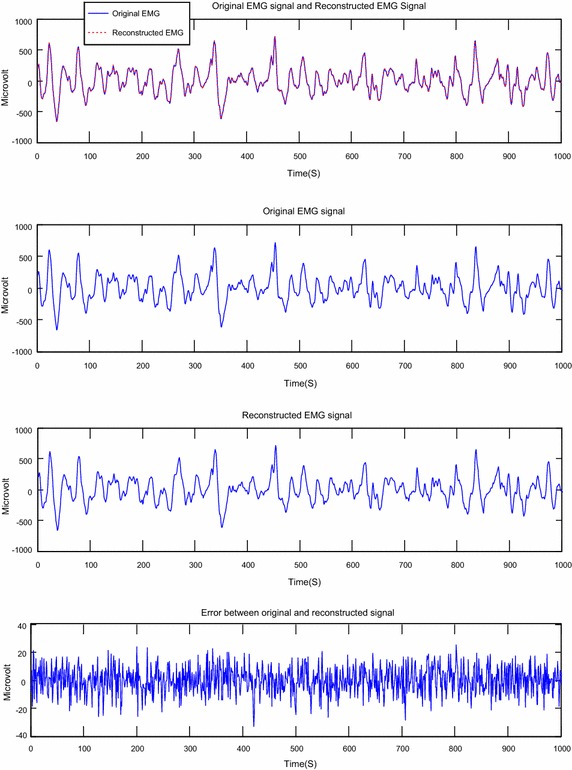
Reconstruction of EMG signal called Kheir1 by DWT; CF = 73.77; PRD = 0.38; SNR = 48.42

**Fig. 7:**
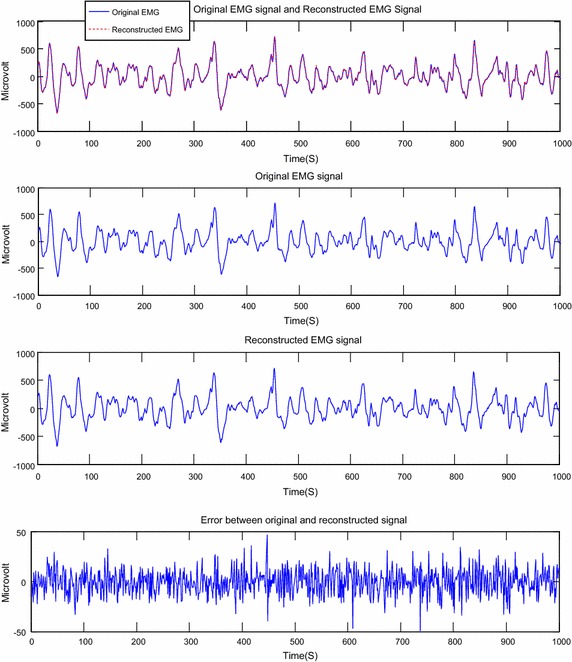
Reconstruction of EMG signal called Kheir1 by DCT; CF = 74.85; PRD = 0.59; SNR = 44.46

**Fig. 8:**
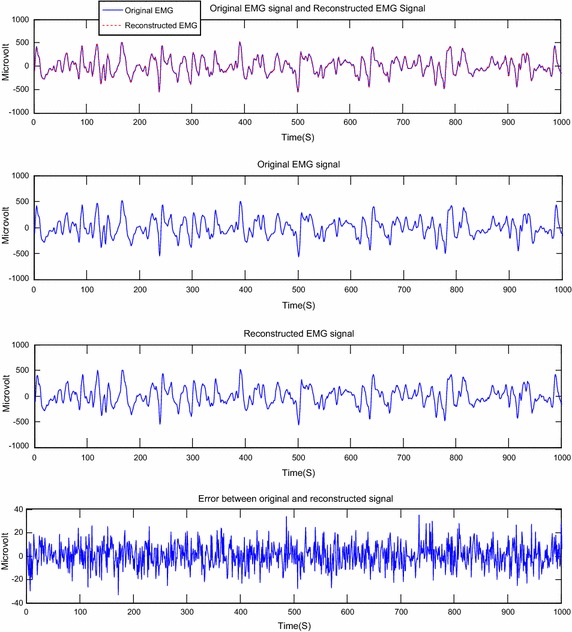
Reconstruction of EMG signal called Kheir2 by DWT: CF = 73.63; PRD = 0.41 %; SNR = 47.79

**Fig. 9:**
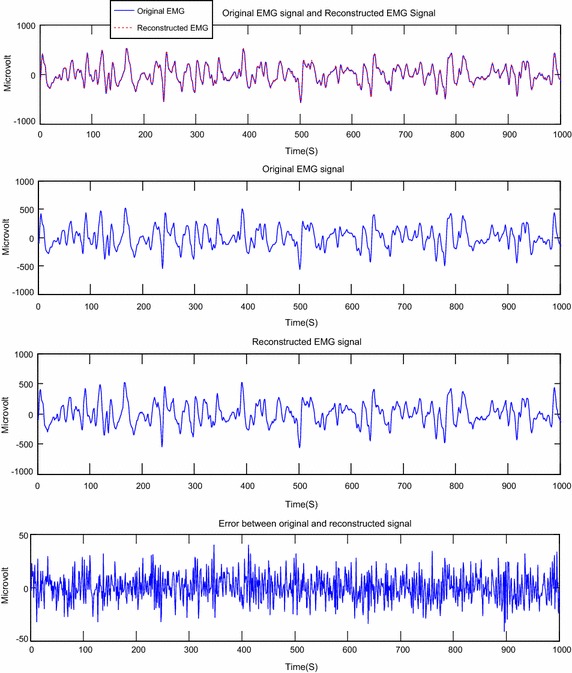
Reconstruction of EMG signal called Kheir2 by DCT; CF = 74.80; PRD = 0.59; SNR = 44.61

**Fig. 10:**
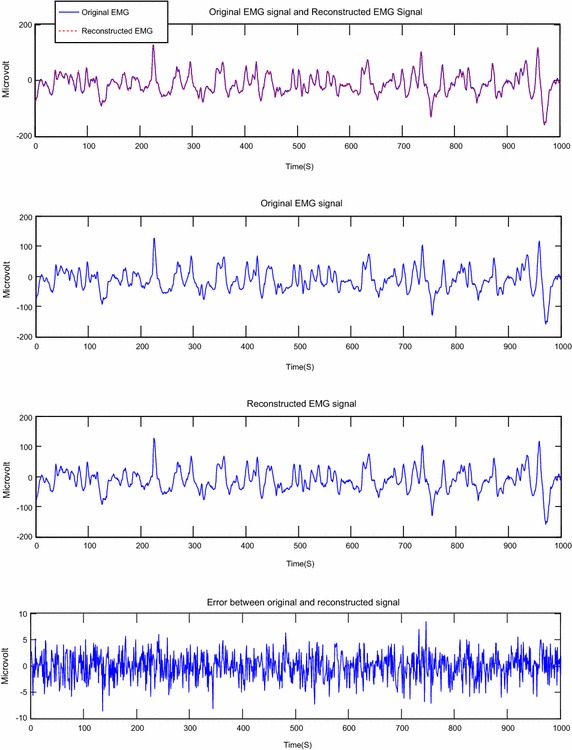
Reconstruction of EMG signal called Jouve3 by DWT; CF = 73.82; PRD = 0.10 %; SNR = 59.72

**Fig. 11:**
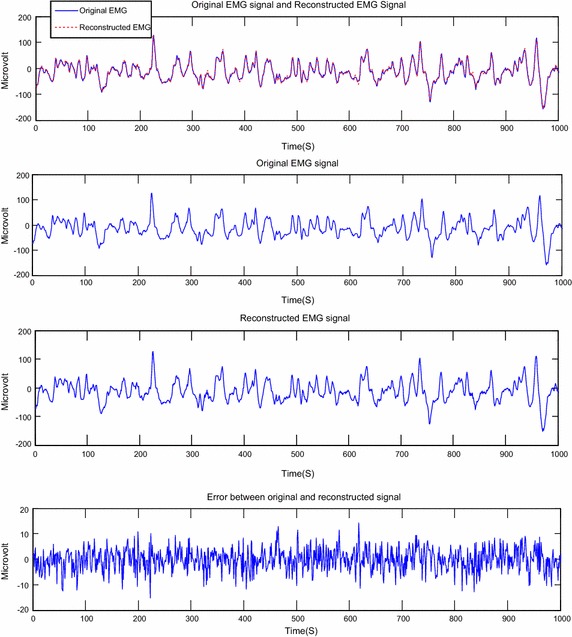
Reconstruction of EMG signal called Jouve3 by DCT; CF = 75.04; PRD = 0.20; SNR = 53.93

The Figs. 6, 7, 8, 9, 10 and 11 present examples of segments of the original signal, of the reconstructed signal and the error signal obtained (difference between original and reconstructed signals) for different methods transform and different EMG signals.

The Tables 1, 2, 3, 4, 5 and 6 show the results obtained on three actuals EMG signals called Kheir1, Kheir2 and Jouve3 for different methods transform and Table 7 shows the comparison of our new compression approach method using wavelet transform or discrete cosine transform and other methods in the literature.

**Table 1:** Obtained results for Kheir1 (with DCT)

CF (%)	PRD (%)	SNR (dB)
87.35	3.22	29.84
81.15	1.30	37.72
74.85	0.59	44.46
68.55	0.30	50.19
62.15	0.16	55.68
55.76	0.08	61.55
49.36	0.042	67.36
43.07	0.02	73.74
36.71	0.01	79.42
11.42	0.0006	103.65

**Table 2:** Obtained results for Kheir2 (with DCT)

CF (%)	PRD (%)	SNR (dB)
87.20	3.01	30.43
80.95	1.28	37.86
74.80	0.59	44.61
68.31	0.30	50.34
61.86	0.16	55.94
55.51	0.08	61.82
49.21	0.04	67.41
42.87	0.02	73.62
36.47	0.01	79.50
11.23	0.0006	103.52

**Table 3:** Obtained results for Jouve3 (with DCT)

CF (%)	PRD (%)	SNR (dB)
87.35	1.30	37.71
81.20	0.42	47.51
75.04	0.2	53.93
68.60	0.10	59.67
62.25	0.04	65.32
55.71	0.026	71.46
49.41	0.013	77.20
43.01	0.0066	83.55
36.71	0.0033	89.53
11.32	0.0002	113.22

**Table 4:** Obtained results for Kheir1 (with DWT)

CF (%)	PRD (%)	SNR (dB)
86.23	1.45	36.80
80.02	0.71	43.00
73.77	0.38	48.42
67.52	0.20	54.01
61.23	0.10	59.89
54.88	0.051	65.80
48.53	0.02	71.86
42.23	0.012	77.80
35.93	0.0061	84.16
10.74	0.0003	108.11

**Table 5:** Obtained results for Kheir2 (with DWT)

CF (%)	PRD (%)	SNR (dB)
86.08	1.58	36.02
79.83	0.77	42.31
73.63	0.41	47.79
67.43	0.21	53.49
61.13	0.11	59.37
54.88	0.05	65.46
48.53	0.02	71.56
42.23	0.01	77.40
35.92	0.006	83.63
10.74	0.0004	107.80

**Table 6:** Obtained results for Jouve3 (with DWT)

CF (%)	PRD (%)	SNR (dB)
86.13	0.4	47.85
79.98	0.18	54.61
73.82	0.10	59.72
67.48	0.052	65.62
61.18	0.027	71.27
54.88	0.013	77.41
48.58	0.0069	83.16
42.23	0.0034	89.13
35.93	0.0016	95.45
10.79	0.0001	119.57

**Table 7:** Comparison of the results (CF, PRD)

Compression factor (%)	70	75	80	85
Norris et al. (2001) for Kheir1	3.90	4.12	5.20	8.02
Berger et al. (2006) for Kheir1	2.57	2.63	3.85	7.01
Berger et al. (2007) for Kheir1	1.79	1.80	2.24	3.13
Filho et al. (2008) for Kheir1	1.21	1.75	2.64	4.18
Trabuco et al. (2014) for Kheir1				
DEA	–	–	3.83	4.82
DLA	2.00	2.29	2.91	4.40
DSR	1.90	2.05	2.39	3.58
RHT	1.85	2.10	2.22	3.31
Proposed method				
Kheir1				
DCT	0.30	0.59	1.30	3.22
DWT	0.20	0.38	0.71	1.45
Kheir2				
DCT	0.30	0.59	1.28	3.01
DWT	0.21	0.41	0.77	1.58
Jouve3				
DCT	0.10	0.2	0.42	1.30
DWT	0.05	0.10	0.18	0.4

## Discussion

The two compression approach methods using DCT and DWT have been implemented. The aim in this article is finding the best transform (DWT or DCT) which is adapted to vector quantization to compress the EMG signals for our new compression approach. About Fig. 4, it appears that PRD increases with compression factor while SNR decreases. Reconstruction quality is good if the PRD is close to zero. This figure shows that wavelet transform whatever the EMG signal, produces an error rate (PRD) maximum of 1.60 %, whereas the discrete cosine transform gives maximum PRD of about 3.4 %.

From Fig. 5, the method by discrete wavelet transform is found to be more efficient than the method through discrete cosine transform.

About Table 1, 2, 3, 4, 5 and 6, we note that the discrete cosine transform gives a compression factor slightly above the discrete wavelet transform. However, the discrete wavelet transform gives smaller PRD. Table 7 shows the comparison of our method and other methods in the literature. We note that, the proposed algorithms give the smaller PRD. We can conclude that, our algorithms provide an improvement in terms of PRD.

About the Figs. 6, 7, 8, 9, 10 and 11, we note that, each figure shows the superposition of the original signal and the reconstructed signal for a better assessment of the power of reconstruction of EMG signals by our algorithm. Then the original signal, reconstructed signal and error between original signal and reconstructed signal are represented. The reconstruction of different EMG signals is represented at compression factor of about 74 % for the discrete wavelet transform and the order of 75 % for the discrete cosine transform. But according to the reconstruction error rate, the wavelet transform keep the lowest error rate.

In telemedicine, the challenge is to have higher compression factor while providing a faithful reconstruction (very small error rate) and avoid any deterioration which may cause a fatal error during the diagnosis of the patient (Istepanian and Petrosian 2000).

Although the discrete cosine transform has brought good results, it is less adequate to compress EMG signals by vector quantization compared to the discrete wavelet transform. About this, we note that the discrete wavelet transform is better suited for compression of EMG by vector quantization.

## Conclusion

In this article, we showed that despite of the good results provided by the discrete cosine transform, it is less suitable for compression of EMG by vector quantization compared to the discrete wavelet transform. The wavelet transform remains appropriate for compression of EMG by vector quantization. This laborious demonstration joined the work of Sana and Kaïs (2009) and Guerrero and Mailhes (1997) which also concluded that the compression method through discrete wavelet transform is found to be significantly better than transform discrete cosine. The proposed algorithms for different signals ensured acceptable quality and also the considerable information retention after reconstruction (CF, PRD and visual observation). In this work we have oriented our choice on vector quantization on K-means algorithm. It would be wise to use other algorithms of vector quantization combined with wavelet transform.
